# Analysis of Lymphocyte Subpopulations and Cytokines in COVID-19-Associated Pneumonia and Community-Acquired Pneumonia

**DOI:** 10.1155/2021/6657894

**Published:** 2021-06-12

**Authors:** Guohong Liu, Xianghu Jiang, Xiaojiao Zeng, Yunbao Pan, Haibo Xu

**Affiliations:** ^1^Department of Radiology, Zhongnan Hospital of Wuhan University, Wuhan University, Wuhan, Hubei, China; ^2^Department of Laboratory Medicine, Zhongnan Hospital of Wuhan University, Wuhan University, Wuhan, Hubei, China

## Abstract

**Background:**

The 2019 novel coronavirus SARS-CoV-2 caused large outbreaks of COVID-19 worldwide. COVID-19 resembles community-acquired pneumonia (CAP). Our aim was to identify lymphocyte subpopulations to distinguish between COVID-19 and CAP.

**Methods:**

We compared the peripheral blood lymphocytes and their subsets in 296 patients with COVID-19 and 130 patients with CAP. Parameters for independent prediction of COVID-19 were calculated by logistic regression.

**Results:**

The main lymphocyte subpopulations (CD3^+^CD4^+^, CD16^+^CD56^+^, and CD4^+^/CD8^+^ ratio) and cytokines (TNF-*α* and IFN-*γ*) of COVID-19 patients were significantly different from that of CAP patients. CD16^+^CD56^+^%, CD4^+^/CD8^+^ratio, CD19^+^, and CD3^+^CD4^+^ were identified as predictors of COVID-19 diagnosis by logistic regression. In addition, the CD3^+^CD4^+^counts, CD3^+^CD8^+^ counts, andTNF-*α* are independent predictors of disease severity in patients.

**Conclusions:**

Lymphopenia is an important part of SARS-CoV-2 infection, and lymphocyte subsets and cytokines may be useful to predict the severity and clinical outcomes of the disease.

## 1. Introduction

COVID-19 is a newly emerging disease with high infection rates, unclear pathogenesis, rapid disease progression, and relatively high incidence of mortality. COVID-19 has affected many countries, with the World Health Organization (WHO) reporting 122536880 confirmed cases and 2703780 deaths up to March21, 2021, globally [1].

Most of the early reports are classified cases as COVID-19 based on the clinical case definition, but specific laboratory confirmation could be made following recognition of SARS-CoV-2 as the pathogen [2]. As the COVID-19 epidemic surges across the globe, researchers are struggling to understand a key epidemiological puzzle—what percentage of infected people have mild or no symptoms and may pass the virus on to others. Some preliminary and detailed estimates of these clandestine cases suggest that they may account for about 60% of all infections [3]. The symptoms COVID-19 appears to cause are similar to other causes of community-acquired pneumonia (CAP), such as fever, cough, shortness of breath, dyspnoea, chest tightness, and diarrhea [4, 5]. Distinguishing COVID-19 from other causes of CAP is one of the main challenges of the COVID-19 outbreak. Our group and others have previously reported numerous hematological abnormalities in COVID-19 [5–8]. Prominent amongst the abnormalities is lymphopenia; although, lymphocyte subsets have not been reported in most studies.

In this study, lymphocyte subsets were examined in a cohort of 296 COVID-19 patients and 130 CAP patients. The present study is aimed at evaluating the ability of lymphocyte subsets and cytokines for distinguishing COVID-19 from CAP.

## 2. Materials and Methods

### 2.1. Patients and Data Collection

The 296 COVID-19 patients presented to our hospital from Feb1, 2020, to Mar10, 2020. All patients were laboratory confirmed to be SARS-CoV-2 infected by real-time RT-PCR. The CAP group consisted of 130 patients who visited our hospital from January 2019 to November 2019. The inclusion criteria included the following: (a) pneumonia was defined as pulmonary infiltration and one or more of the following symptoms: fever (body temperature ≥ 38.0°C), cough with or without sputum discharge, dyspnea, or changes in breathing sounds by auscultation; (b) complete patient records of lymphocyte subsets; and (c) hospital patients. The exclusion criteria were as follows: (a) patients lacking data on clinical lymphocyte subsets and (b) outpatient patients. During that hard time, 244 (82.4%) COVID-19 patients took antiviral medicine at home, but none of the COVID-19 patients received immunomodulating drugs before visiting the hospital. All the COVID-19 patients received blood sampling after the onset of symptoms. The clinical data collected from the patients was approved by the Ethics Committee of Zhongnan Hospital of Wuhan University. The Ethics Committee waived written informed consent for emerging infectious diseases.

### 2.2. Lymphocyte Subpopulation Test

**Figure 1:**
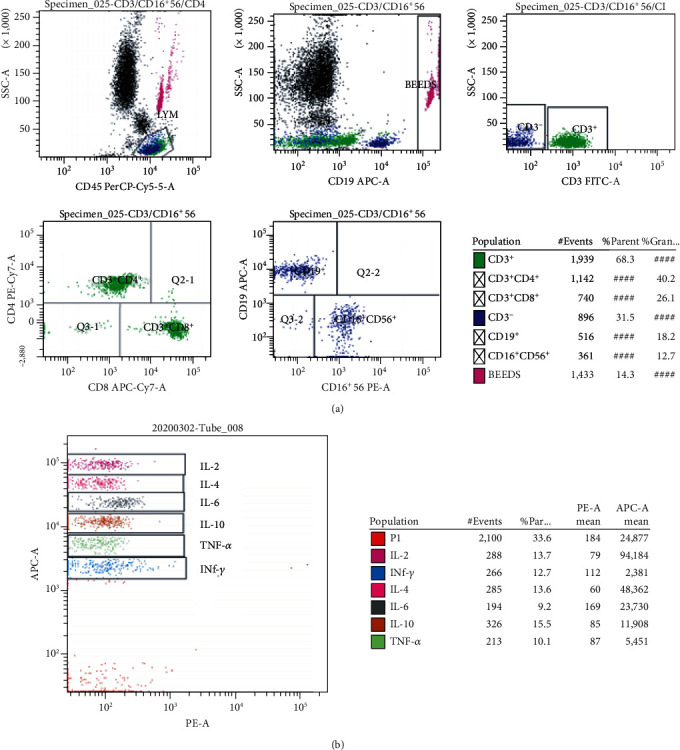
Flow cytometry analysis template to detect the CD3^+^, CD3^+^CD4^+^, CD3^+^CD8^+^, CD19^+^, and CD16^+^CD56^+^ cells (a) and 6 kinds of cytokines (b) in one tube simultaneously.

Fasting whole blood from every patient was collected aseptically by venipuncture into ethylenediamine tetraacetic acid (EDTA) collection tubes for the quantification of the main lymphocyte subpopulations. Whole blood was incubated with BD Multitest 6-color TBNK reagent and then lysed with BD FACS™ lysing solution. Lymphocyte subpopulations were acquired and analyzed with BD FACSCanto clinical software. The BD Multitest 6-color TBNK reagent contains the following antibodies to identify and count different lymphocyte subsets: CD3 FITC was used for T lymphocyte identification, CD16 and CD56 PE for NK lymphocyte identification, CD45 PerCP-Cy™5.5 for lymphocyte population identification, CD4 PE-Cy™7 for T-helper/inducer lymphocyte identification and CD19 APC B lymphocyte identification, and CD8 APC-Cy7 for inhibitory/toxic T lymphocyte subset identification. The final results can be easily observed in the flow cytometry template we established (Figure 1(a)).

Generally, we pipette 20 *μ*L of BD Multitest 6-color TBNK reagent into the bottom of the BD Trucount tube and then pipette 50 *μ*L of well-mixed, anticoagulated whole blood into the bottom of the tube. Cap the tube and vortex gently to mix followed by incubating for 15 minutes in the dark at room temperature (20°C–25°C). We add 450 *μ*L of 1X BD FACS lysing solution to the tube and incubate the tube for 15 minutes in the dark at room temperature. The samples were then analyzed on the flow cytometer. Absolute counts are calculated by BD FACSCanto clinical software using the following formula.

(1) $$\begin{array}{c} \frac{\#\text{events in cell population}}{\#\text{events in absolute count bead region}}\times \frac{\#\text{beads}/\text{test}*}{\text{test volume}}=\text{cell population absolute count} \end{array}$$

∗This value is found on the BD Trucount tube foil pouch label and can vary from lot to lot.

### 2.3. Cytokine Analysis

This method involved Multiplex Cytometric Bead Array (CBA) for quantitative analysis of 6 kinds of cytokines, including tumor necrosis factor-alpha (TNF-*α*), interferon-gamma (IFN-*γ*), IL-6, IL-2, IL-4, and IL-10. The multiplex CBA was performed according to the manufacturer's instructions. Briefly, 25 *μ*L serum was mixed with an equal volume of capture beads and incubated with 25 *μ*L of PE-binding antibodies in the dark at room temperature for 2.5 hours. The beads were then centrifuged at 200 g for 5 min, and the supernatant was gently aspirated and resuscitated with phosphate buffer brine (PBS) (100 *μ*L). The CBA was addressed in a flow cytometer (BD) and analyzed by clinical software. The final result can be easily observed in the flow cytometry template we have established (Figure 1(b)).

### 2.4. Statistical Analysis

Statistical analysis was performed using SPSS (Version 22.0, SPSS, Inc., Chicago, IL, USA). Statistical analysis for the results was performed using the Student *t*-test. A *p* value <0.05 was considered statistically significant.

## 3. Results

**Figure 2:**
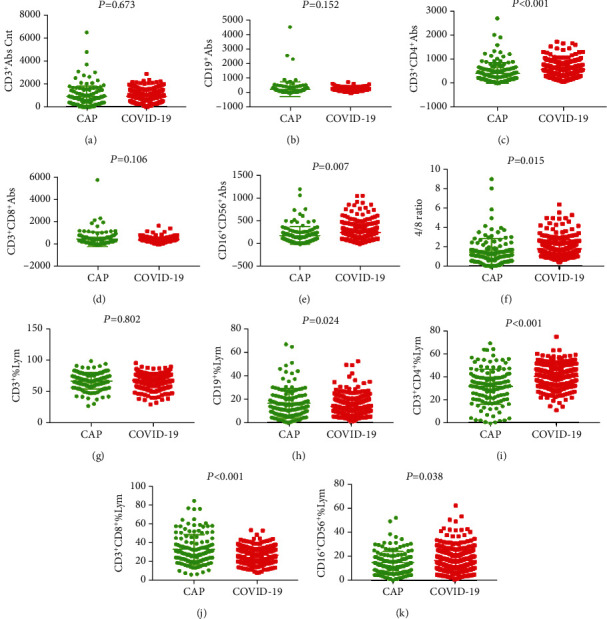
General characteristics of lymphocyte subpopulations between CAP patients and COVID-19 patients.

A total of 296 COVID-19 patients (152male vs. 144female), with a mean age of 53 years and 130 CAP patients (80male vs. 50female), with a mean age of 50 years that were hospitalized at Zhongnan Hospital of Wuhan University, were enrolled in the study. Among the 130 CAP patients, 76 (58.5%) patients had bacterial pneumonia, 31 (23.8%) had viral pneumonia, 5 (3.8%) patients had fungal pneumonia, 5 (3.8%) patients had mycoplasma pneumonia, and 13 (10.0%) patients had pneumocystosis or other infection. The mean values of lymphocyte subpopulations indexes in COVID-19 patients and CAP patients were demonstrated in Table 1 and Figures 1 and 2. The mean values of CD19^+^% and CD3^+^CD8^+^% in COVID-19 patients were significantly lower than those in patients with CAP. The mean values of CD3^+^CD4^+^, CD16^+^CD56^+^, CD4/CD8 ratio, CD3^+^CD4^+^%, and CD16^+^CD56^+^% in COVID-19 patients were significantly higher than those in patients with CAP. The mean values of CD3^+^, CD19^+^, and CD3^+^CD8^+^ were not significantly different between the COVID-19 group and CAP group.

**Figure 3:**
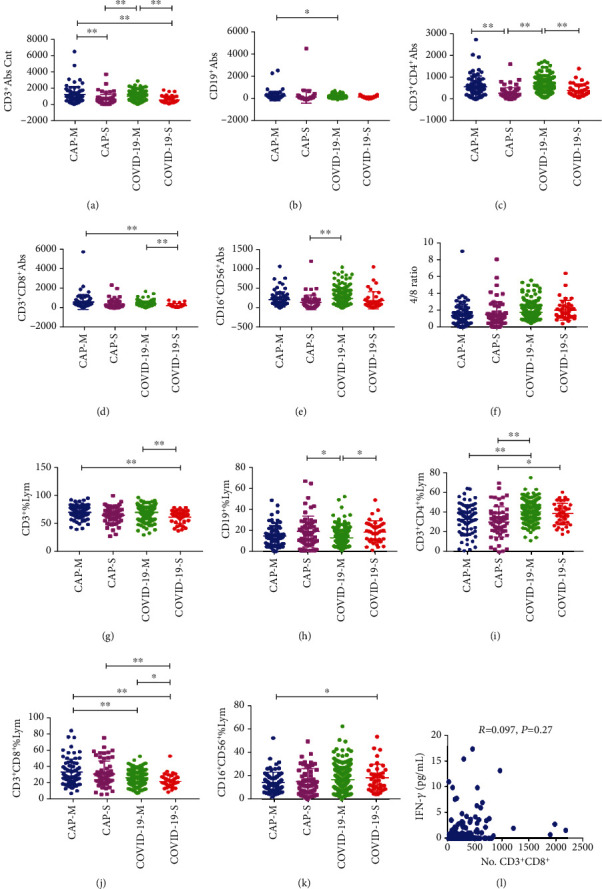
General characteristics of lymphocyte subpopulations in mild CAP patients (CAP-M), severe CAP patients (CAP-S), mild COVID-19 patients (COVID-19-M), and severe COVID-19 patients (COVID-19-S). ^∗^*p* < 0.05, ^∗∗^*p* < 0.01.

In the mild group (71 CAP and 257 COVID-19), COVID-19 patients showed decreased CD19^+^, CD3^+^CD8^+^%, and increased CD3^+^CD4^+^%, compared with that of CAP patients. Similarly, in the severe patients (59 CAP and 39 COVID-19), COVID-19 patients had increased CD3^+^CD4^+^% and decreased CD3^+^CD8^+^%, compared with that of CAP patients (Figure 3).

The proportion of patients with abnormal lymphocyte subpopulations is shown in Table 2. Both COVID-19 patients and CAP patients had lymphopenia. A higher percentage of CAP patients showed reduced CD3^+^, CD3^+^CD4^+^, reduced CD16^+^CD56^+^, reduced CD4^+^/CD8^+^ ratio, increased CD19^+^%, and normal CD3^+^CD4^+^% compared with the COVID-19 patients. Logistic regression analysis showed that laboratory indicators could independently distinguish between COVID-19 and CAP. The ORs of the factors to predict COVID-19 versus CAP were demonstrated in Table 3.The CD16^+^CD56^+^%, CD4^+^/CD8^+^ ratio, CD19^+^, and CD3^+^CD4^+^ independently discriminating COVID-19 from CAP. In addition, the CD3^+^CD4^+^ and CD3^+^CD8^+^ counts are independent predictors of disease severity in the COVID-19 group and the combined COVID-19 and CAP group (Table 4).

**Figure 4:**
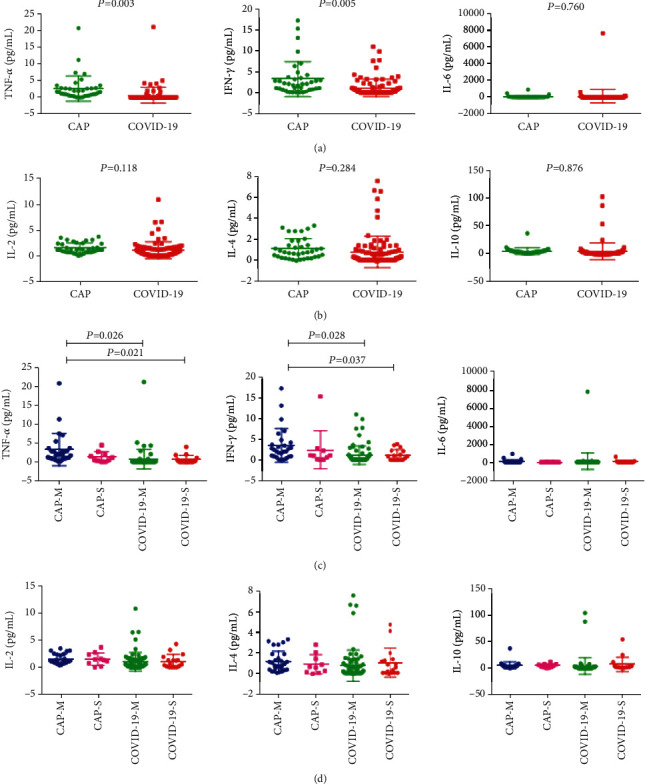
General characteristics of cytokines in patients with mild CAP (CAP-M), severe CAP (CAP-S), mild COVID-19 (COVID-19-M), and severe COVID-19 (COVID-19-S). ^∗^*p* < 0.05, ^∗∗^*p* < 0.01.

In this study, we also analyzed 6 kinds of cytokines data in 92 COVID-19 patients and 38 CAP patients (Figures 1(b) and 4). TNF-*α* and IFN-*γ* had lower level in COVID-19 patients compared with the CAP patients. However, we found that IFN-*γ* levels were not correlated with CD8^+^ cytotoxic T lymphocytes in COVID-19 patients based on the database analysis, suggesting that decreased IFN-*γ* is not caused by CD8^+^ cytotoxic T lymphocytes (Figure 3(l)). As to IL-6, IL-2, IL-4, and IL-10, we did not find significant difference between the two groups. Logistic regression analysis revealed that TNF-*α* independently discriminate disease severity in COVID-19 patients (Table 5).

## 4. Discussion

The interaction between COVID-19 and the immune system is complex. In the current study, lymphocyte subsets and cytokines were examined in COVID-19 and CAP patients. A significant proportion of COVID-19 patients had reduced lymphocyte subpopulations. This study confirmed the lymphopenia observed in most of the other series of COVID-19 cases [5–7]. The data discussed here extend these observations, showing that the CD3^+^CD4^+^ and CD16^+^CD56^+^ lymphocyte counts were higher but the TNF-*α* and IFN-*γ* were lower in COVID-19 patients compared with those of CAP patients.

The most commonly used lymphocyte subsets are currently detected, including T lymphocytes (CD3^+^), B lymphocytes (CD19^+^), NK cells (CD16^+56+^), helper T lymphocytes (CD3^+^CD4^+^), and suppressor T lymphocytes (CD3^+^CD8^+^). Percentages and absolute counts of T and B lymphocytes and the ratio of helper/inducer versus suppressor/cytotoxic T cells provide valuable information on immune status for a number of patient conditions [7]. Helper T lymphocytes cells can help B cells secrete antibodies and regulate the immune response of other T cells [9]. It can release IL-2, IFN-*γ*, IL-4, and other cytokines and activate macrophages and NK cells [10]. Suppressor T lymphocytes cells often exhibit cytotoxic activity and are the major cytotoxic effector cells. As the main immune cells of the body's natural immune system, NK lymphocytes have been shown to be cytotoxic to certain tumors and viruses [11, 12]. Secreted antibodies and mediator humoral immune response are the major functions of B lymphocytes. Activated B lymphocytes can secrete antigens and induce T cell immunity.

Lymphopenia is an important part of COVID-19, and the lymphocyte count may be used to predict the severity of the disease and clinical outcome. Total and subset lymphopenia also occurs in other human coronavirus SARS infections [13]. Experimental coronavirus 229E infections resulted in lymphopenia in humans [14]. The lymphopenia in COVID-19 may be attributed to direct viral invasion and destruction of lymphocytes from SARS-CoV-2. However, studies suggest that the human receptor for COVID-19 could be angiotensin-converting enzyme 2 (ACE2) [15]. ACE2 is the functional cellular receptor for the SARS-CoV-2 but does not express in B or T lymphocytes [16, 17]. This suggests that lymphopenia in COVID-19 is not directly infected and destroyed by SARS-CoV-2 and requires further study.

Other possible explanations for lymphopenia are lymphocyte isolation in the lung where SARS-CoV-2 damage is most pronounced [18], or cytokine-mediated altered lymphocyte transport [7]. Coronavirus 229E can induce apoptosis of monocytes/macrophages in vitro [19]. It is not clear whether different strains of SARS-CoV-2 induce lymphocyte apoptosis. SARS-CoV-2-induced immunosuppression may be predisposed to secondary infection, especially in severely ill patients, and it remains to be determined whether there are any long-term effects on humoral or cell-mediated immunity.

Our findings demonstrated that lymphocyte subsets features, especially CD16^+^CD56^+^%, CD4^+^/CD8^+^ ratio, CD19^+^, and CD3^+^CD4^+^ independently predicted the differentiation of COVID-19 and CAP. The CD3^+^CD4^+^, CD3^+^CD8^+^ counts, and TNF-*α* are independent predictors of disease severity. Thus, detection of lymphocyte subsets and cytokines provides new insights into the pathogenesis of COVID-19 and CAP, which is helpful to understand the immune function of patients and is worthy of popularization and application.

## Figures and Tables

**Table 1:** Laboratory values of COVID-19 patients and CAP patients.

Variable	CAP (*n* = 130)	COVID-19 (*n* = 296)	*p*
Age (years)	50 (32-72)	53 (41-64)	0.230
Gender (M/F)	80/50	152/144	0.057
CD3^+^	936.37 (322.75-1187.5)	973.11 (596.75-1274)	0.673
CD19^+^	248.5 (55.25-285.5)	184.43 (92-246)	0.152
CD3^+^CD4^+^	426.42 (133.25-580.25)	579.1 (334-766.5)	<0.001
CD3^+^CD8^+^	462.48 (123.75-540.75)	368.9 (212.75-488)	0.106
CD16^+^CD56^+^	183.98 (64.25-227.5)	238.59 (107.75-304.25)	0.007
4/8 ratio	1.47 (0.57-1.73)	1.81 (1.19-2.21)	0.015
CD3^+^% lym	67.5 (60.1-77.19)	67.8 (62.43-74.87)	0.802
CD19^+^% lym	16.69 (7.41-22.32)	14 (8.6-17.8)	0.024
CD3^+^CD4^+^% lym	31.79 (21.81-39.57)	40.38 (33.72-46.49)	<0.001
CD3^+^CD8^+^% lym	31.87 (21.18-39.42)	25.7 (20-31.4)	<0.001
CD16^+^CD56^+^% lym	14.6 (7.21-19.4)	16.75 (9.68-22.2)	0.038

**Table 2:** Abnormal laboratory results for COVID-19 patients and CAP patients.

Variate	COVID-19 (*n* = 296)	CAP (*n* = 130)	*p*

CD3^+^			<0.001
Normal	185 (62.5%)	56 (43.1%)	
High	0 (0%)	2 (1.5%)	
Low	111 (37.5%)	72 (55.4%)	

CD19^+^			0.032
Normal	81 (27.4%)	35 (26.9%)	
High	0 (0%)	3 (2.3%)	
Low	215 (72.6%)	92 (70.8%)	

CD3^+^CD4^+^			<0.001
Normal	217 (73.3%)	59 (45.4%)	
High	0 (0%)	1 (0.8%)	
Low	79 (26.7%)	70 (53.8%)	

CD3^+^CD8^+^			0.216
Normal	140 (47.3%)	55 (42.3%)	
High	0 (0%)	1 (0.8%)	
Low	156 (52.7%)	74 (56.9%)	

CD16^+^CD56^+^			0.008
Normal	129 (43.6%)	39 (30%)	
Low	167 (56.4%)	91 (70%)	

4/8 ratio			<0.001
Normal	169 (57.1%)	58 (44.6%)	
High	88 (29.7%)	26 (20%)	
Low	39 (13.2%)	46 (35.4%)	

CD3^+^% lym			0.679
Normal	153 (51.7%)	70 (53.8%)	
High	139 (47%)	57 (43.8%)	
Low	4 (1.4%)	3 (2.3%)	

CD19^+^% lym			0.004
Normal	164 (55.4%)	66 (50.8%)	
High	15 (5.1%)	19 (14.6%)	
Low	117 (39.5%)	45 (34.6%)	

CD3^+^CD4^+^% lym			<0.001
Normal	106 (35.8%)	69 (51.1%)	
High	189 (63.9%)	45 (36.7%)	
Low	1 (0.3%)	16 (12.2%)	

CD3^+^CD8^+^% lym			<0.001
Normal	264 (89.2%)	88 (67.7%)	
High	15 (5.1%)	34 (26.2%)	
Low	17 (5.7%)	8 (6.2%)	

CD16^+^CD56^+^% lym			0.018
Normal	231 (78%)	88 (67.7%)	
High	16 (5.4%)	5 (3.8%)	
Low	49 (16.6%)	37 (28.5%)	

**Table 3:** Multivariate predictors of COVID-19 versus CAP.

Variate	OR	95% CI	*p*
CD3^+^CD4^+^% lym	0.951	0.637-1.422	0.808
CD19^+^% lym	1.048	0.805-1.363	0.729
CD3^+^% lymcnt	0.871	0.54-1.402	0.569
CD16^+^CD56^+^% lym	1.338	1.032-1.736	0.028
CD3^+^CD8^+^% lym	1.334	0.886-2.009	0.167
4/8 ratio	1.538	1.166-2.028	0.002
CD19^+^	0.743	0.566-0.975	0.032
CD3^+^CD4^+^	1.822	1.417-2.343	<0.001
CD16^+^CD56^+^	1.102	0.838-1.449	0.488
CD3^+^	1.174	0.776-1.778	0.447
CD3^+^CD8^+^	0.834	0.636-1.094	0.19

**Table 4:** Multivariate predictors of lymphocyte subsets on disease severity.

Variate	COVID-19			CAP and COVID-19		
	OR	95% CI	*p*	OR	95% CI	*p*
4/8 ratio	0.901	0.531-1.527	0.698	1.083	0.762-1.539	0.657
CD16^+^CD56^+^% lym	0.851	0.519-1.395	0.522	1.068	0.774-1.474	0.688
CD16^+^CD56^+^ abs	1.212	0.744-1.975	0.441	1.133	0.811-1.583	0.463
CD19^+^% lym	0.874	0.552-1.386	0.568	0.911	0.677-1.226	0.539
CD19^+^ abs	0.622	0.387-1	0.05	0.744	0.528-1.049	0.092
CD3^+^% lymcnt	0.585	0.287-1.194	0.141	0.864	0.542-1.378	0.54
CD3^+^abs cnt	0.873	0.425-1.796	0.713	1.277	0.821-1.988	0.278
CD3^+^CD4^+^% lym	0.938	0.402-2.186	0.881	0.93	0.597-1.448	0.747
CD3^+^CD4^+^ abs	2.046	1.328-3.151	0.001	2.515	1.862-3.397	<0.001
CD3^+^CD8^+^% lym	1.041	0.546-1.984	0.904	1.161	0.767-1.757	0.48
CD3^+^CD8^+^ abs	2.218	1.288-3.819	0.004	1.539	1.126-2.103	0.007

**Table 5:** Multivariate predictors of cytokines on disease severity.

Variate	COVID-19			CAP and COVID-19		
	OR	95% CI	*p*	OR	95% CI	*p*
IFN-*γ*	1.04	0.76-1.423	0.807	1.017	0.865-1.195	0.841
IL-10	1.187	0.996-1.414	0.055	1.01	0.982-1.038	0.503
IL-2	0.626	0.203-1.926	0.414	1.021	0.461-2.26	0.96
IL-4	1.794	0.971-3.315	0.062	1.265	0.823-1.942	0.283
IL-6	0.998	0.996-1	0.092	0.999	0.997-1.001	0.306
TNF-*α*	0.361	0.138-0.941	0.037	0.874	0.694-1.101	0.254

## Data Availability

The data used to support the findings of this study are included within the article.
